# Triglycerides and low HDL cholesterol predict coronary heart disease risk in patients with stable angina

**DOI:** 10.1038/s41598-021-00020-3

**Published:** 2021-10-20

**Authors:** Chiara Caselli, Raffaele De Caterina, Jeff M Smit, Jonica Campolo, Mohammed El Mahdiui, Rosetta Ragusa, Alberto Clemente, Tiziana Sampietro, Aldo Clerico, Riccardo Liga, Gualtiero Pelosi, Silvia Rocchiccioli, Oberdan Parodi, Arthur Scholte, Jhuani Knuuti, Danilo Neglia, EVINCI and SMARTool

**Affiliations:** 1 Institute of Clinical Physiology CNR, Via G. Moruzzi 1, Pisa, Italy; 2 Cardiovascular Department, Fondazione Toscana G. Monasterio, Via G. Moruzzi 1, Pisa, Italy; 3 Cardiothoracic and Vascular Department, Azienda Ospedaliero-Universitaria Pisana, Via Roma, 67, Pisa, Italy; 4 Department of Cardiology, Leiden University Medical Centre, Albinusdreef 2, Leiden, The Netherlands; 5 Institute of Clinical Physiology CNR, ASST Grande Ospedale Metropolitano Niguarda, P.Zza Ospedale Maggiore, 3, Milan, Italy; 6 Sant’Anna School of Advanced Studies, Piazza Martiri Della Libertà, 33, Pisa, Italy; 7 PET Center, Turku University Hospital and University of Turku, Kiinamyllynkatu 4-8, Turku, Finland

**Keywords:** Biomarkers, Cardiology, Risk factors

## Abstract

We assessed whether high triglycerides (TG) and low high-density lipoprotein cholesterol (HDL-C) levels, expressed by an increased TG/HDL-C ratio, predict coronary atherosclerotic disease (CAD) outcomes in patients with stable angina. We studied 355 patients (60 ± 9 years, 211 males) with stable angina who underwent coronary computed tomography angiography (CTA), were managed clinically and followed for 4.5 ± 0.9 years. The primary composite outcome was all-cause mortality and non-fatal myocardial infarction. At baseline, the proportion of males, patients with metabolic syndrome, diabetes and obstructive CAD increased across TG/HDL-C ratio quartiles, together with markers of insulin resistance, hepatic and adipose tissue dysfunction and myocardial damage, with no difference in total cholesterol or LDL-C. At follow-up, the global CTA risk score (HR 1.06, 95% confidence interval (CI) 1.03–1.09, *P* = 0.001) and the IV quartile of the TG/HDL-C ratio (HR 2.85, 95% CI 1.30–6.26, *P* < 0.01) were the only independent predictors of the primary outcome. The TG/HDL-C ratio and the CTA risk score progressed over time despite increased use of lipid-lowering drugs and reduction in LDL-C. In patients with stable angina, high TG and low HDL-C levels are associated with CAD related outcomes independently of LDL-C and treatments.

**Trial registration.** EVINCI study: ClinicalTrials.gov NCT00979199, registered September 17, 2009; SMARTool study: ClinicalTrials.gov NCT04448691, registered June 26, 2020.

## Introduction

In patients with suspected or known coronary artery disease (CAD) a standard lipid profile including total cholesterol (Total-C), high density lipoprotein cholesterol (HDL-C), low-density lipoprotein cholesterol (LDL-C), and triglycerides (TG) should be assessed^1–3^.Multiple studies, including either general populations or patients with known CAD, have shown that all the individual components of the lipid profile are markers of coronary heart disease (CHD) risk^3–5^. Major attention has been dedicated to total and LDL cholesterol levels based on evidence that substantial reduction in these parameters are effective in reducing CHD events and the progression of coronary atherosclerosis^1–3^. More recently, TGs and not-LDL cholesterol are emerging as relevant determinants of global atherosclerotic cardiovascular disease (ASCVD) risk which may persist despite aggressive LDL-C lowering^4–6^. There is evidence that high TGs and low HDL-C characterize a specific cardio-metabolic profile known as atherogenic dyslipidemia, which is independent of LDL-C, correlates with the metabolic syndrome, insulin resistance, and incident diabetes and is associated with ASCVD risk in general populations^7–10^. This condition may specifically influence CHD events and response to statin treatment in high-risk patients^11–15^. An elevated TG/HDL-C ratio, which has been proposed as an easily obtainable marker of atherogenic dyslipidemia, has been associated with adverse long-term CHD outcomes and all-cause mortality in secondary prevention studies performed in high-risk gender-specific^12,13^ or country-specific^14^ cohorts with known CAD, as well as in patients presenting with acute coronary syndromes^15^.

Whether the cardio-metabolic profile described by the TG/HDL-C ratio is progressive over time and is associated with CHD events and progression of coronary atherosclerosis in patients with intermediate-low risk is not known. Such patients represent a large population of either males and females, with stable angina and without known CAD increasingly referred for diagnostic screening in countries with advanced or growing economies. Despite intense resource utilization, both in the diagnostic and in the therapeutic process, the efficacy of current management to improve outcomes in this population is debated^16^. Thus, the identification of a new specific condition associated with higher CHD risk and disease progression not tackled by current treatments is a relevant unmet need^17^.

Accordingly, aim of the present study was to characterize the cardiometabolic disorder described by higher TG/HDL-C ratio and to assess whether it predicts CHD events and progression of coronary atherosclerosis in a prospectively enrolled European population of patients with stable angina referred to non-invasive diagnostic screening and managed with currently indicated conservative or invasive treatments.

## Results

### Baseline clinical characteristics of the study population

**Figure 1:**
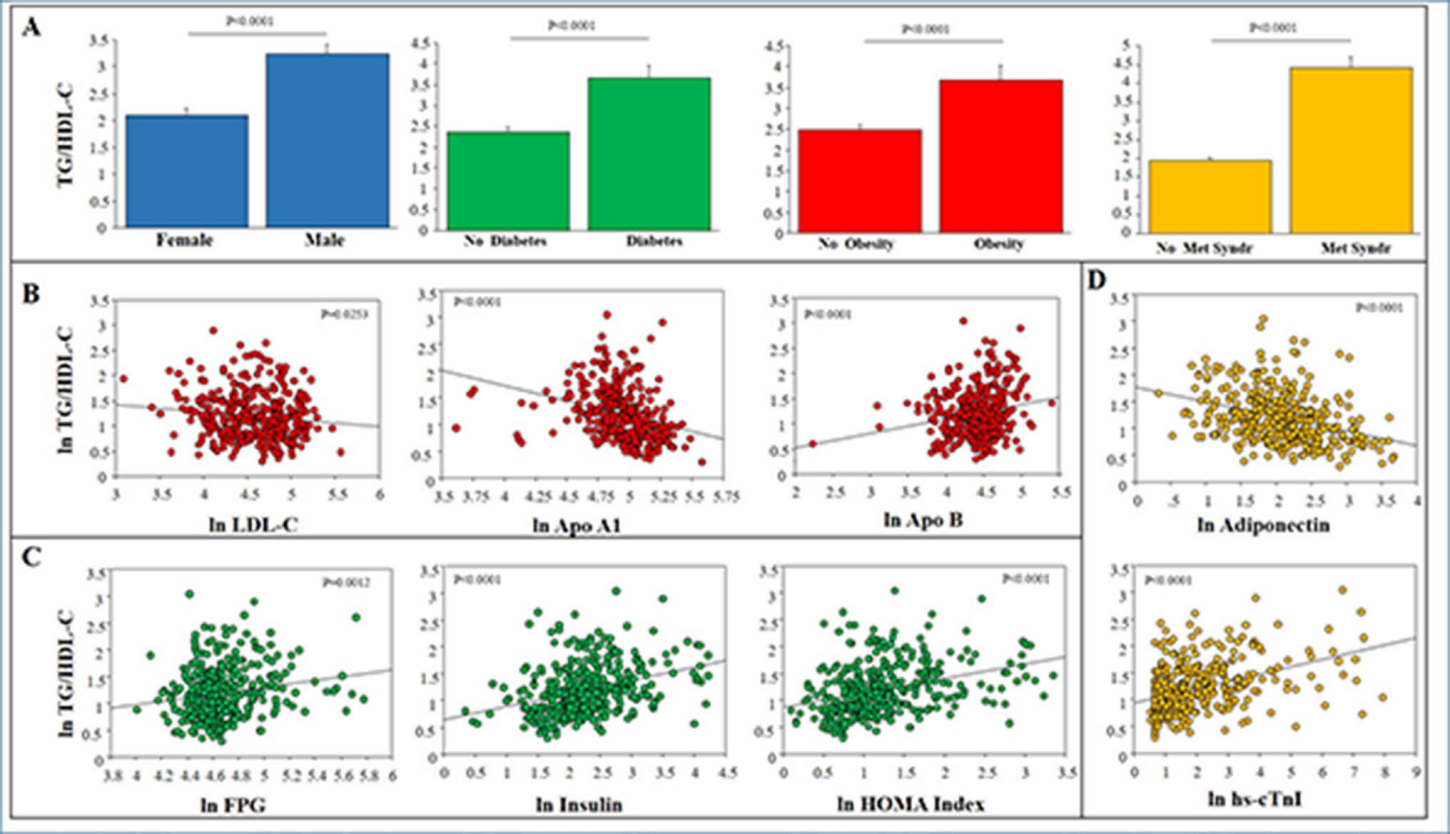
TG/HDL-C ratio and cardio-metabolic risk. Relationship between baseline TG/HDL-C ratio, specific cardiovascular risk factors (Panel **A**), and relevant bio-humoral variables (Panel **B**, **C**, **D**).

In the overall study population, the mean value of TG/HDL ratio was 2.78 ± 2.37 (mean ± SD) and the median value was 2.095 (interquartile range, IQR: 2.079). Demographic and clinical characteristics, cardiovascular risk factors and medication use are detailed in Table 1. In the whole population, mean age was 60 ± 9 years and 59% of patients were men. Family history of CAD was present in 33% of patients and the majority of the population had atypical chest pain (60%), hypercholesterolemia (61%) and hypertension (60%). Diabetes was diagnosed in 112 (31%) and metabolic syndrome in 118 (33%) patients. In the different TG/HDL ratio quartiles, the frequency of male gender, diabetes, smoking habit and of the metabolic syndrome significantly increased from the I quartile to the IV quartile. Male patients and patients with diabetes, obesity (BMI > 30) and metabolic syndrome showed significantly higher TG/HDL ratio as compared with female patients or patients without diabetes, obesity and the metabolic syndrome, respectively (Fig. 1, Panel A). A significant increase across quartiles was found for BMI, the use of beta-blockers, diuretics and glucose-lowering agents.

**Table 1:** Clinical characteristics of the study population and of groups defined by TG/HDL-C quartiles. [Continuous variables are presented as mean ± standard deviation, categorical variables as absolute N and (%); * = P value only for comparison of Quartile IV vs. Quartile I].

	Study population n = 355	Quartile I < 1.305 n = 89	Quartile II 1.305–2.095 n = 89	Quartile III 2.129–3.384 n = 88	Quartile IV > 3.384 n = 89	*P* value for trend
**Demographics**						
Age, years	60 ± 9	60 ± 8	61 ± 9	61 ± 9	59 ± 9	ns
Male gender	211 (59)	35 (39)	47 (53)	61 (69)	68 (76)	< 0.0001
**Clinical characteristics**						
Typical angina	87 (25)	21 (23)	26 (29)	18 (21)	22 (25)	ns
Atypical angina	213 (60)	55 (62)	53 (60)	53 (60)	52 (58)	
Non-anginal chest pain	55 (15)	13 (15)	10 (11)	17 (19)	15 (17)	
LVEF%	60 ± 8	61 ± 8	61 ± 8	60 ± 9	59 ± 9	ns
**Cardiovascular risk factors**						
Family history of CAD	118 (33)	26 (29)	31 (35)	31 (35)	30 (34)	ns
Diabetes	112 (31)	18 (20)	20 (22)	32 (36)	42 (48)	0.0002
Hypercholesterolemia	216 (61)	50 (56)	55 (62)	56 (64)	55 (62)	ns
Hypertension	266 (64)	54 (61)	61 (69)	54 (61)	57 (65)	ns
Smoking	78 (22)	13 (15)	19 (21)	18 (20)	28 (31)	0.0076*
BMI, kg/m^2^	27.7 ± 4.1	26.2 ± 3.6	27.3 ± 3.8	28.3 ± 4.5	29.1 ± 3.9	< 0.0001
Metabolic syndrome	118 (33)	7 (8)	16 (18)	29 (33)	66 (74)	< 0.0001
**Medications**						
Beta-blockers	141 (40)	35 (39)	32 (36)	28 (32)	46 (52)	0.0430
Calcium channel blockers	47 (13)	9 (10)	15 (17)	12 (13)	11 (12)	ns
ACE inhibitors	112 (32)	25 (28)	24 (27)	27 (31)	36 (40)	ns
ARBs	55 (15)	12 (13)	21 (24)	11 (12)	11 (12)	ns
Diuretics	60 (17)	4 (4)	19 (21)	14 (16)	23 (26)	0.0011
Anti-diabetic	72 (20)	11 (12)	15 (17)	17 (19)	29 (33)	0.0059
Statins	192 (54)	46 (52)	47 (53)	47 (53)	52 (58)	ns
Aspirin	221 (62)	51 (57)	57 (64)	53 (60)	60 (67)	ns
Anti-coagulants	6 (2)	2 (2)	1 (1)	0 (0)	3 (3)	ns

### Baseline bio-humoral profiles

The comparison of bio-humoral measurements across TG/HDL-C quartiles is reported in Table 2. The lipid profile was characterized by progressively higher TG, remnant-C, Apolipoprotein B (Apo-B) and Apo-B/Apolipoprotein A1 (Apo-A1) and lower HDL-C, Apo-A1 and Proprotein Convertase subtilisin/kexin type 9 (PCSK9) levels without significant differences in Total-C, LDL-C and lipoprotein(a) (Lp(a)). Biomarkers of abnormal glucose metabolism and hepatic dysfunction (alanine aminotransferase (ALT) and gamma-glutamyl transferase (GGT)) progressively increased across TG/HDL-C quartiles. Adiponectin progressively decreased and high sensitivity Troponins progressively increased in patients from the I Quartile to the IV Quartile.

**Table 2:** Bio-humoral data of the study population and of groups defined by TG/HDL-C quartiles. [Continuous variables are presented as mean ± standard deviation; # = LDL-C was not calculated in 3 patients with TG > 400 mg/dL in the IV quartile].

Biomarkers	Study population n = 355	Quartile I < 1.305 n = 89	Quartile II 1.305–2.095 n = 89	Quartile III 2.129–3.384 n = 88	Quartile IV > 3.384 n = 89	*P* value for trend
**Lipid metabolism**						
Total cholesterol, mg/dL	180 ± 47	184 ± 45	182 ± 50	172 ± 47	180 ± 47	ns
LDL-C, mg/dL#	104 ± 38	106 ± 36	108 ± 39	103 ± 39	99 ± 39	ns
HDL-C, mg/dL	52 ± 17	66 ± 17	56 ± 14	46 ± 11	39 ± 9	< 0.0001
Total/HDL-C	3.7 ± 1.1	2.84 ± 0.58	3.30 ± 0.56	3.87 ± 1.01	4.67 ± 1.17	< 0.0001
Remnant-C	24 ± 15	12 ± 4	19 ± 5	24 ± 7	42 ± 17	< 0.0001
Triglicerides, mg/dL	123 ± 77	61 ± 17	92 ± 23	119 ± 31	219 ± 89	< 0.0001
Triglicerides/HDL-C	2.78 ± 2.37	0.96 ± 0.25	1.67 ± 0.23	2.63 ± 0.41	10.05 ± 0.46	< 0.0001
Apo A1, mg/dL	143 ± 32	158 ± 35	149 ± 31	136 ± 26	128 ± 26	< 0.0001
Apo B, mg/dL	86 ± 27	80 ± 22	85 ± 25	84 ± 29	96 ± 29	0.0027
Apo B/Apo A1	0.62 ± 0.22	0.54 ± 0.26	0.57 ± 0.12	0.62 ± 0.16	0.77 ± 0.24	< 0.0001
Lipoprotein (a), mg/dL	21.9 ± 24.3	23.4 ± 27.0	23.8 ± 22.7	23.3 ± 25.5	16.9 ± 21.3	ns
PCSK9, ng/mL	223 ± 136	247 ± 130	220 ± 123	226 ± 129	200 ± 156	0.0125
**Glucose metabolism**						
FPG, mg/dL	111 ± 36	103 ± 20	109 ± 37	116 ± 42	117 ± 38	0.0209
TyG index	8.65 ± 0.63	8.00 ± 0.35	8.46 ± 0.37	8.77 ± 0.41	9.35 ± 0.46	< 0.0001
Insulin, mUI/mL	11.3 ± 11.0	7.2 ± 6.3	9.9 ± 8.9	12.4 ± 11.8	15.5 ± 13.7	< 0.0001
HOMA-IR index	3.3 ± 4.0	1.9 ± 1.9	2.9 ± 3.5	3.8 ± 4.7	4.7 ± 4.7	< 0.0001
**Hepatic function**						
AST, IU/L	24 ± 9	25 ± 9	24 ± 9	24 ± 9	25 ± 10	ns
ALT, IU/L	21 ± 11	19 ± 11	19 ± 9	22 ± 13	22 ± 11	0.0117
ALP, IU/L	51 ± 18	50 ± 18	50 ± 18	52 ± 18	52 ± 18	ns
GGT, IU/L	39 ± 30	36 ± 43	35 ± 30	37 ± 15	47 ± 20	< 0.0001
**Inflammation**						
hs-CRP, mg/dL	0.41 ± 1.26	0.48 ± 2.24	0.42 ± 0.83	0.44 ± 0.68	0.32 ± 0.42	ns
Interleukin 6, ng/L	1.35 ± 2.63	1.33 ± 3.81	1.21 ± 1.32	1.55 ± 2.37	1.29 ± 2.46	ns
**Adipocytokines**						
Leptin, ng/mL	10.4 ± 11.0	10.5 ± 9.1	12.0 ± 14.4	9.1 ± 9.1	10.1 ± 10.4	ns
Adiponectin, mg/mL	9.6 ± 6.5	13.8 ± 8.7	9.0 ± 5.1	9.0 ± 5.4	6.8 ± 3.8	< 0.0001
**Myocardial Damage**						
hs-cTnT, ng/L	8.0 ± 6.2	7.0 ± 5.7	7.4 ± 6.3	9.0 ± 6.8	8.5 ± 6.0	0.0043
hs-cTnI, ng/L	52.5 ± 233.6	23.4 ± 154.6	42. 6 ± 300.5	46.8 ± 158.5	97.2 ± 278.3	< 0.0001
NT-proBNP, ng/L	134.8 ± 223.2	133.9 ± 172.8	104.1 ± 127.2	168.6 ± 325.6	133.0 ± 217.8	ns

In the overall population, the TG/HDL-C ratio was weakly related with LDL-C while it was strongly and negatively related with ApoA1 and adiponectin as well as strongly and positively related with Apo B and hsTnI (Fig. 1, Panel B, C, D). Excluding patients with diabetes, the same relationships were maintained with the exception of LDL-C and FPG, which were no longer related with the TG/HDL-C ratio (Fig. S1of Supplementary Material).

### Association of TG/HDL-C ratio with CAD and outcomes

Baseline coronary CTA, stress imaging, invasive procedures and outcome data are reported in Table 3 for the overall population and in groups defined by TG/HDL-C ratio quartiles. While the frequency of obstructive CAD at CTA and of significant inducible ischemia at stress imaging was not significantly different across TG/HDL-C quartiles, the number of total coronary plaques, and in particular of mixed/non-calcified plaques as well as the CTA score had a trend to increase across TG/HDL-C quartiles and were significantly higher in patients in the IV as compared with the I quartile. Patients in the IV quartile were more frequently referred to ICA, had more frequent invasive diagnosis of obstructive CAD, and were more frequently submitted to early revascularization.

**Table 3:** Imaging and Outcomes in the study population and in groups defined by TG/HDL-C quartiles. [Continuous variables are presented as mean ± standard deviation, categorical variables as absolute N and (%); * = P value only for comparison of Quartile IV vs. Quartile I].

	Study Population n = 355	Quartile I < 1.305 n = 89	Quartile II 1.305–2.095 n = 89	Quartile III 2.129–3.384 n = 88	Quartile IV > 3.384 n = 89	*P* value for trend
**Baseline CTA**						
Obstructive CAD	104 (29)	19 (21)	30 (34)	27 (31)	28 (31)	ns
N. of plaques	3.87 ± 3.82	3.01 ± 3.56	4.03 ± 4.12	4.18 ± 3.53	4.24 ± 3.94	0.0321*
N. of calcified plaques	0.88 ± 1.72	0.72 ± 1.49	0.71 ± 1.65	1.18 ± 1.92	0.90 ± 1.77	ns
N. of mixed/non-calcified	2.99 ± 3.50	2.29 ± 3.17	3.33 ± 3.88	3.00 ± 3.24	3.34 ± 3.59	0.0462*
CTA score	11.53 ± 11.04	9.28 ± 9.98	12.04 ± 11.94	12.22 ± 10.63	12.62 ± 11.37	0.0432*
**Baseline Stress Imaging**						
Significant ischemia	77 (22)	18 (20)	16 (18)	28 (32)	15 (17)	ns
**Baseline Invasive Procedures**						
ICA Performed	234 (66)	52 (62)	55 (62)	65 (74)	62 (70)	0.0444*
Obstructive CAD	89 (25)	14 (16)	19 (27)	19 (21)	28 (31)	0.0135*
Early Revascularization	66 (19)	11 (12)	16 (18)	17 (19)	22 (25)	0.0339*
**Outcome**						
Composite outcome end-point	25 (7)	2 (2)	8 (9)	3 (4)	12 (13)	0.0114
Death from any cause	8 (2)	2 (2)	1 (1)	2 (2)	3 (3)	ns
Non-fatal MI	17 (5)	0 (0)	7 (8)	1 (1)	9 (10)	0.0024
Late Revascularization	25 (7)	3 (3)	9 (10)	5 (6)	8 (9)	ns

**Figure 2:**
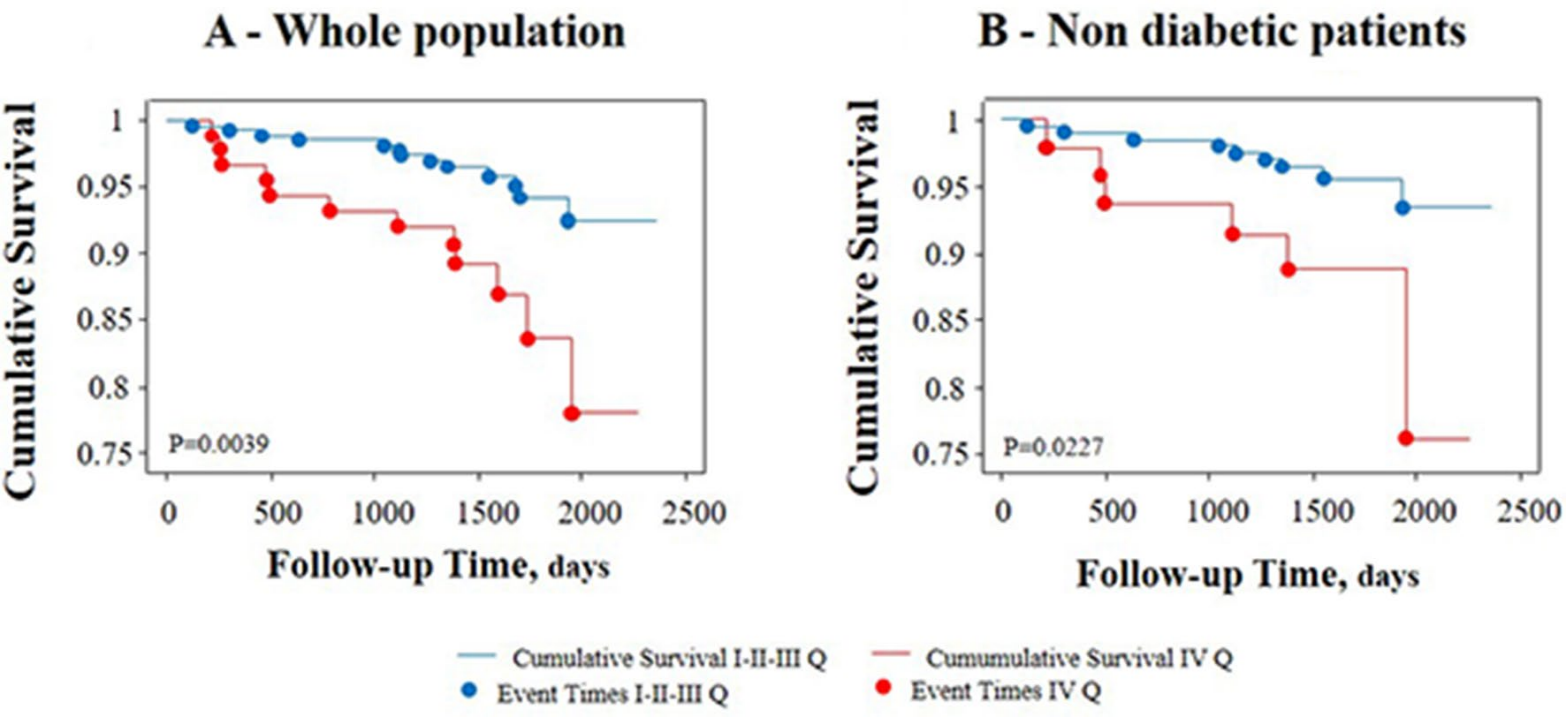
Survival analysis. Unadjusted Kaplan–Meier estimates of the primary composite endpoint according to TG/HDL-C quartiles (IV vs I-II-III) in the whole population (Panel **A**) or after exclusion of diabetic patients (Panel **B**).

The median value of the clinical follow-up was 4.5 ± 0.9 years. During the follow-up, 25 patients (7%) experienced a major AE, including death in 8 patients (2%) (cardiac death in only one patient) and non-fatal myocardial infarction in 17 patients (5%). The primary composite outcome was progressively more frequent from the I to the IV quartile of TG/HDL-C ratio, mainly due to more frequent occurrence of non-fatal myocardial infarction (Table 3). The Kaplan–Meier survival plots showed that event-free survival was significantly worse in patients in the highest TG/HDL-C ratio quartile (Fig. 2A). The same occurred including only patients without diabetes (Fig. 2B).

At univariate Cox analysis, among all clinical, risk and treatment variables, only male gender was significantly associated with the primary composite outcome (Table S1). Among biomarkers, the TG/HDL ratio (both continuous values and the IV quartile), HDL-C, TG, remnant-C, ApoA1, TyG index, and hs-cTnT were associated with outcome while other biomarkers of lipid or glucose metabolism were not (Table S2). Among imaging variables and invasive procedures, all the coronary disease parameters from CTA, CTA score and obstructive CAD at ICA were also significantly associated with outcome (Table S3).

At multivariable Cox analysis (Table 4), the highest TG/HDL-C ratio (IV quartile) and the CTA score were the only predictors of the primary composite outcome independently of all the other variables associated with outcome at the univariable analysis (Model 1). The inclusion of ApoA1 into the model slightly decreased the association of the TG/HDL-C ratio with the outcome but still TG/HDL-C ratio (IV quartile), the CTA score and ApoA1 remained independent predictors of prognosis (Model 2).

**Table 4:** Association of TG/HDL (IVQ) with the composite outcome end-point at multivariable Cox analysis.

	Outcome					
	Model 1			Model 2		
	HR	95% CI	P value	HR	95%CI	P value
**TG/HDL-C ratio**	2.850	1.297–6.263	0.0091	2.518	1.092–5.807	0.0303
**CTA score**	1.060	1.026–1.095	0.0004	1.066	1.028–1.106	0.0006
**Apo A1**	–	–	–	0.215	0.067–0.691	0.0098

### Association of TG/HDL-C ratio with progression of coronary atherosclerosis

A second coronary CTA follow-up scan was performed in 154 patients with an interscan period of 6.2±1.4 years. Changes in clinical characteristics, treatments, bio-humoral profiles and CTA risk score from the time of the 1st to the time of the 2nd CTA scan are summarized in Table 5. Typical angina was less frequent at the time of the second scan while the use of medications was more frequent with the exception of diuretics. The use of statins had increased from 51 to 64% of patients and of anti-diabetics from 18 to 29%. While LDL-C significantly decreased from baseline to follow-up (associated with opposite changes in HDL-C and PCSK9), there was a significant increase in TG levels, remnant-C, the TG/HDL-C ratio, together with indexes of insulin resistance (HOMA, Tyg index), systemic inflammation (IL-6) and endothelial activation (VCAM1). Similarly, the CTA score worsened at follow-up (from 11.02 ± 10.46 to 12.94 ± 9.93, *P*<0.0001) indicating a progression of coronary atherosclerosis and associated risk.

**Table 5:** Changes from baseline of relevant variables in 154 patients who performed a follow-up CTA. [Continuous variables are presented as mean ± standard deviation, categorical variables as absolute N and (%)].

	Study population N = 154		
	Baseline	Follow up	*P* value
**Clinical characteristics**			
Age, years	61 ± 8	68 ± 8	< 0.0001
Male gender	85 (55)	85 (55)	–
Typical angina	47 (30)	13 (8)	0.0563
BMI	27.53 ± 3.60	27.21 ± 3.38	ns
**Medications**			
Beta-blockers	69 (45)	73 (47)	< 0.0001
Calcium channel blockers	12 (8)	34 (22)	< 0.0001
ACE Inhibitors	50 (32)	66 (43)	< 0.0001
ARBs	26 (17)	26 (17)	ns
Diuretics	27 (17)	23 (15)	< 0.0001
Anti-diabetic	28 (18)	44 (29)	< 0.0001
Statins	78 (51)	98 (64)	< 0.0001
Aspirin	93 (60)	101 (66)	ns
Anti-coagulants	1 (1)	2 (1)	ns
**Lipids Metabolism**			
Total cholesterol, mg/dL	181 ± 48	178 ± 47	ns
LDL-C, mg/dL	106 ± 40	94 ± 41	0.0004
HDL-C, mg/dL	52 ± 17	57 ± 18	< 0.0001
Total/HDL-C	3.7 ± 1.1	3.3 ± 0.9	< 0.0001
Remnant-C, mg/dL	22 ± 11	27 ± 16	0.0001
Triglicerides, mg/dL	112 ± 59	145 ± 102	< 0.0001
Triglicerides/HDL-C	2.49 ± 1.96	2.96 ± 3.02	0.0027
PCSK9, ng/mL	226 ± 160	247 ± 80	< 0.0001
**Glucose metabolism**			
FPG, mg/dL	109 ± 27	109 ± 28	ns
Insulin, μUI/mL	11.8 ± 11.3	11.2 ± 11.4	ns
HOMA-IR index	3.2 ± 3.9	3.3 ± 4.5	< 0.0001
TyG index	8.57 ± 0.56	8.80 ± 0.59	< 0.0001
**Hepatic function**			
AST, IU/L	24 ± 9	24 ± 8	ns
ALT, IU/L	20 ± 9	20 ± 9	ns
ALP, IU/L	49 ± 19	53 ± 19	ns
GGT, IU/L	35 ± 18	31 ± 17	0.0016
**Inflammation**			
hs-CRP, mg/dL	0.40 ± 0.72	0.30 ± 0.42	0.0907
Interleukin 6, ng/L	1.02 ± 1.27	1.81 ± 3.84	< 0.0001
**Endothelial activation**			
ICAM1, ng/mL	192 ± 73	197 ± 83	ns
VCAM1, ng/mL	529 ± 151	600 ± 160	< 0.0001
**Myocardial damage**			
hs-cTnT, ng/L	7.56 ± 5.29	7.68 ± 5.55	ns
**CTA**			
CTA Score	11.02 ± 10.46	12.94 ± 9.93	< 0.0001

**Figure 3:**
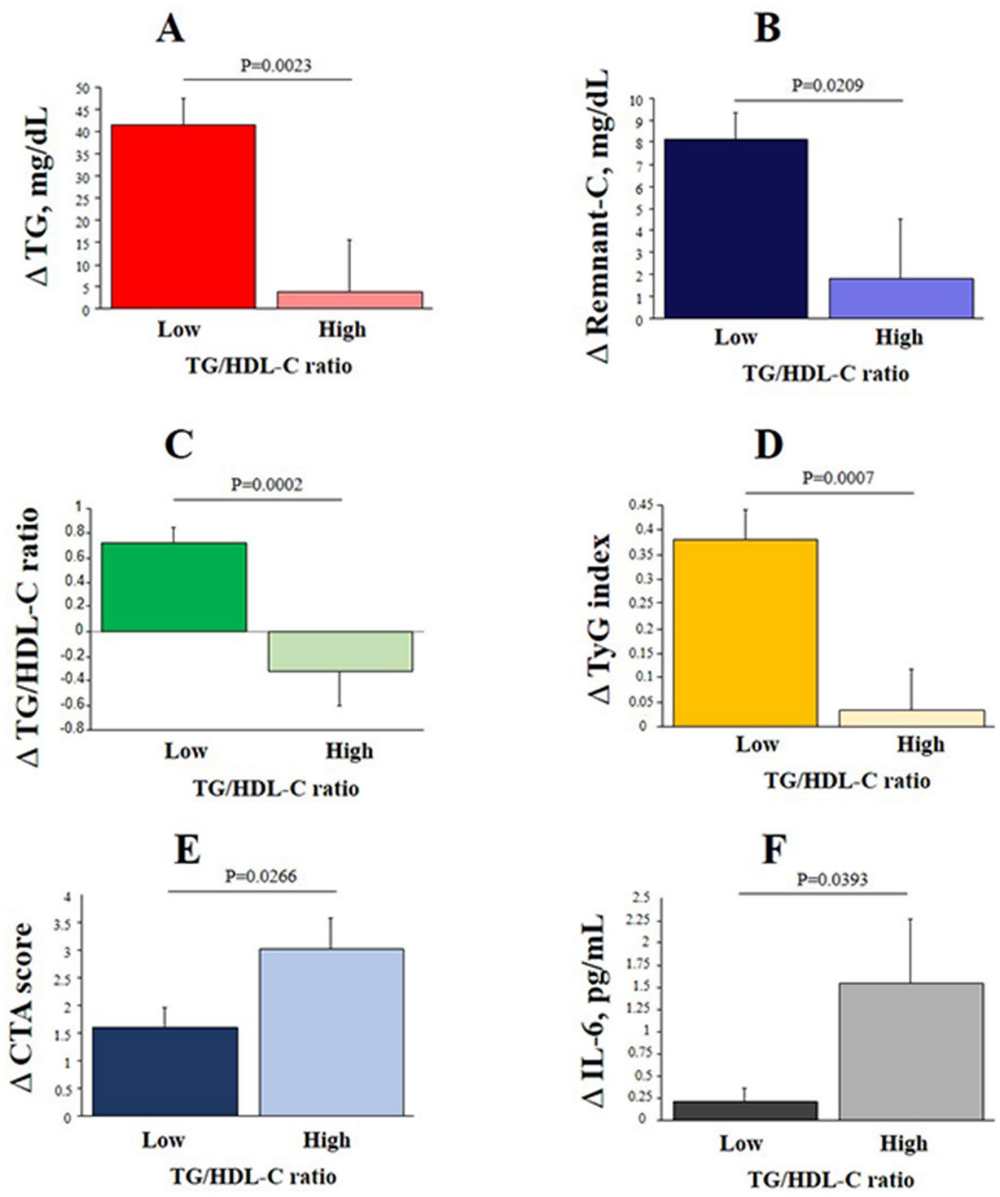
Association of TG/HDL-C ratio with progression of cardio-metabolic and CAD risk. Bar graphs represent ∆ changes (follow-up minus baseline) for the TGs (**A**), remnant-C (**B**), TG/HDL-C ratio (**C**), TyG index (**D**), CTA score (**E**), and IL-6 (**F**) levels from baseline to follow-up in two groups defined by baseline TG/HDL-C ratio below (low) or above (high) the median value.

Changes from baseline to follow-up in the two subgroups of patients defined by their baseline TG/HDL-C ratio below (N = 88) or above (N = 66) the median value (2.095) are reported in Table S4. The specific cardio-metabolic risk profile, expressed by TG/HDL-C ratio and associated variables (TG, remnant-C and the TyG index), progressed in patients with lower TG/HDL-C at baseline. In patients with higher TG/HDL-C at baseline both the ratio and the associated variables remained stably high (Table S4 and Fig. 3, panels A-D). The CTA score, Il-6 and VCAM-1 increased in both groups (Table S4) but changes in the CTA score and IL-6 were more prominent in patients with higher TG/HDL-C already at baseline (Fig. 3, panels E–F).

## Discussion

The TG/HDL-C ratio describes a specific lipid and glucose metabolic profile which was able to predict CHD events and progression of coronary atherosclerosis in patients with stable angina and without known disease enrolled in a prospective, multicenter international study. In this population at intermediate-low risk, patients with a higher TG/HDL-C ratio had clinical and bio-humoral features of atherogenic dyslipidemia, insulin resistance, metabolic syndrome and diabetes together with a higher coronary atherosclerosis burden at CTA. Both a higher TG/HDL-C ratio and the coronary CTA score were additional predictors of worse outcome, independently of other cardiovascular risk factors, the presence of obstructive CAD or ischemia. This residual risk was not modified by conventional medical treatments or revascularization procedures. In those patients who were submitted to a clinical and bio-humoral re-evaluation and a follow-up CTA scan over a 6 years period, the specific cardio-metabolic disorder identified by higher values of TG/HDL-C ratio at baseline remained stable over time while it progressed in the other patients. The coronary atherosclerotic process also evolved in both groups, as indicated by increase in markers of endothelial activation, systemic inflammation and of the coronary CTA score, despite increasing use of medications and lowering of LDL-C levels. Nevertheless, most of these changes were more prominent in patients who had a higher TG/HDL-C ratio already at baseline and thus a more prolonged exposure to this specific cardiometabolic risk.

The relationship between TG/HDL-C ratio, the specific cardio-metabolic profile known as atherogenic dyslipidemia and CAD related clinical outcomes has been previously described either in general populations^7–10^ or in high risk patients with known CAD or acute coronary syndromes^11–15^. In particular, in two gender-specific^12^ or country-specific^14^ populations of high-risk patients referred for ICA, the highest values of the TG/HDL-C distribution were associated with twofold increase in all-cause mortality and 1.5- to threefold increase in cardiovascular events during a 5–6 years follow-up, independently of traditional coronary risk factors and angiographic CAD severity. Our results now expand these and other observations in a prospective European multicenter population of intermediate-low risk patients with stable angina, without previous CAD and a low prevalence of obstructive disease (25% at ICA). All-cause mortality and the rate of non-fatal myocardial infarction (2% and 5% at 4.5 years) were much lower than in previous studies. However, also in this population, a higher TG/HDL-C ratio (>3.38, IV quartile) was associated with an almost twofold increase in the risk of non-fatal myocardial infarction and of the composite outcome end-point (all-cause death and non-fatal myocardial infarction). Even more importantly, the prognostic role of higher TG/HDL-C ratio was additive to that of the well-established coronary CTA atherosclerosis risk score (18) and was independent of other cardiovascular risk factors, biomarkers and the presence of more severe symptoms, inducible ischemia and/or obstructive CAD at ICA. The combined model including TG/HDL-C ratio and the CTA score predicted residual CHD risk also independently of revascularization procedures and medical treatments.The addition to the prognostic model of the CTA score attenuated the hazard ratio of TG/HDL-C alone suggesting a causal link between this variable and CAD related outcome. A possible causal relationship between the cardio-metabolic profile, expressed by TG/HDL-C ratio, and residual CHD risk, not tackled by current treatments, was also suggested in the present study by the observations collected in a subgroup of patients who were re-evaluated at follow up receiving a second blood sampling and a second CTA scan. Despite a more frequent use of cardioactive and risk controlling medications, including statins and anti-diabetics, and the improvement of circulating LDL-C levels, the specific lipid and glucose disorder associated with higher TG/HDL-C ratio either remained unaltered or worsened. This behavior was paralleled by an overall increase in the bio-humoral markers of endothelial activation and systemic inflammation and more importantly by a worsening of the coronary atherosclerotic burden described by the CTA score. The effects on systemic inflammation and coronary atherosclerotic risk pattern were even more pronounced in patients with a TG/HDL-C ratio already elevated at baseline who were possibly exposed to an adverse cardio-metabolic condition for longer periods.

The pathophysiological implications of elevated TG and low HDL-C levels, as well as of the associated lipid and glucose profile in atherogenesis have been widely explored and reported^19^ and are consistent with the results of the present study. In the present population, higher TG/HDL-C values were associated with the bio-humoral features of atherogenic dyslipidemia^11^, the metabolic syndrome, insulin resistance^7,12^ and more in general of a diabetic or pre-diabetic state^20^. Patients with higher values of TG/HDL-C ratio had also higher values of remnant-C, which has been recently implicated in atherogenesis, inflammation and prediction of CHD risk^4–6^, and of the TyG index, which has been proposed as a surrogate indicator of “insulin resistance” and related with CHD risk^21,22^ and progression of coronary atherosclerosis^23^. Interestingly, in the present low risk population of patients without previous disease, the TG/HDL-C ratio was a better predictor of CAD related outcomes than either remnant-C or the TyG index. This observation suggests that, at least in these patients, both TGs and HDL-C function may have an interactive role^19,24^. TG-rich lipoproteins carry cholesterol in addition to TGs and this, together with LDL-C is considered to be the atherogenic agent that feeds the development of arterial wall plaques^4,5,25^. Elevated TG and VLDL levels, are associated with lower levels of HDL-C and formation of small, dense HDL and small, dense LDL particles^19^. Cholesteryl ester transfer protein (CETP) catalyzes the transfer of cholesteryl esters (CE) from HDL to ApoB-containing lipoproteins (VLDL and LDL) in exchange for TG. In the context of higher levels of TGs and VLDL, this exchange results in higher proportion of TG-rich small, dense HDL which are catabolized more rapidly, leading to low levels of HDL-C, reduction of reverse cholesterol transport (RCT) and reduced antioxidant and anti-inflammatory properties^24–26^. On the other hand, when TG levels are high, ApoB particles are more TG-enriched. The hydrolysis of the TGs within the TG-rich LDL by hepatic lipase remodels the LDL particles which become smaller and denser and can enter the arterial intima more easily and are more atherogenic. Small, dense LDL particles also bind weakly to the LDL receptor, thus increasing their half-life in the plasma and being more subject to oxidative damage and to subsequent uptake by the macrophage scavenger receptors^19^.Moreover, our patients were characterized by an extensive bio-humoral evaluation. Higher values of TG/HDL-C ratio were associated with lower circulating levels of ApoA1, higher levels of hs-cTnI and, over time, of IL-6 and VCAM1. Thus, the cardiometabolic risk profile defined by TG/HDL-C was associated with a downregulation of endothelial protective mechanisms and an upregulation of systemic and vascular inflammatory processes, translating into progressive coronary atherosclerosis and subclinical myocardial damage^27^. In this context, a reduced functionality of HDL particles might contribute to the coronary atherogenic risk^19,28^. This may explain why, besides the TG/HDL-C ratio and CTA score, circulating ApoA1 levels were the only additional independent predictors of adverse outcome in our population.

We recognize some limitations of the present study.LDL-C was not determined directly but was calculated with the Friedewald formula. It was computed in 352/355 patients since in 3 cases TG levels were higher than 400 mg/dL. The study population was well characterized but was relatively small and included only symptomatic patients with suspected obstructive CAD but at intermediate-low risk. We used a composite outcome end-point since our ability to separately model mortality was limited by the small number of deaths. Cardiac death occurred in only one patient thus only all-cause death could be considered. Lack of statistical significance for some covariates usually associated with risk of death may thus reflect low power rather than lack of prognostic value. The number of patients with repeated CTA scans was not sufficient to estimate the independent association between changes in biomarkers and in specific CTA parameters such as plaque features.Moreover, not all biomarkers were measured at follow-up in this subpopulation, thus changes in some biomarkers could not be assessed. On the other hand, ICAM1 and VCAM1 levels were not available for association analysis with the TG/HDL-C ratio in the entire population. The study included a highly selected population of patients who presented for a clinically indicated diagnostic work-up for stable angina with normal cardiac function. Thus, it is unknown whether our findings extend to a general population of asymptomatic subjects with similar risk factors or to higher risk populations with known acute or chronic coronary syndromes or with cardiac dysfunction and heart failure.

In conclusion, obesity, insulin resistance and metabolic syndrome are emerging conditions in contemporary populations of symptomatic patients with suspected CAD^29^; the present results suggest that the TG/HDL-C ratio is a simple method to identify among these patients, independently of the severity of symptoms or the presence of obstructive disease and inducible ischemia, those individuals with a substantial residual coronary risk not tackled by current treatments. The underlying genetic and molecular pathways are complex and deserve continuing mechanistic research to develop new targeted nutritional, lifestyle and drug strategies beyond the established LDL-C lowering interventions. Some pharmacological approaches are already promising in this context, including omega 3-fatty acids, PCSK9 inhibitors, PPAR-alfa agonists and ATP-citrate lyase inhibitors^30,31^. Most of these drugs have been tested in primary or secondary prevention studies but their efficacy in improving outcome in selected patients with suspected CAD and high cardiometabolic risk, such as those associated with the highest TG/HDL-C ratio has not been established.

## Methods

### Study population

**Figure 4:**
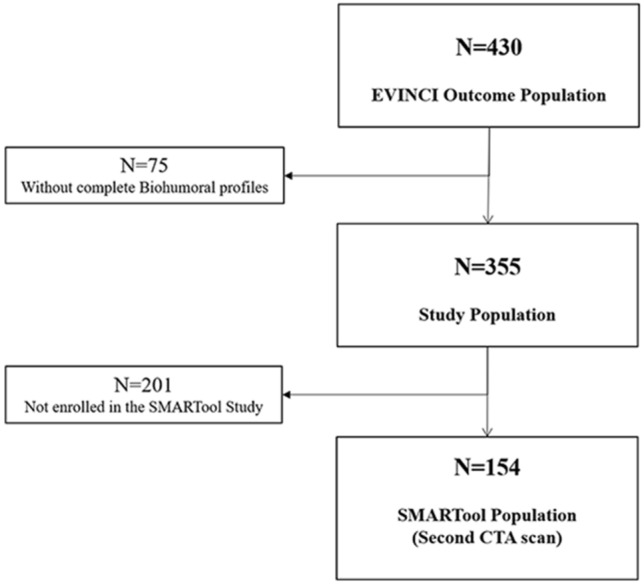
Study flow diagram.

The study population was identified within the EValuation of INtegrated Cardiac Imaging (EVINCI) Outcome study cohort. Design and primary results of the EVINCI studies have been previously published^32,33^. In brief, EVINCI was a multicenter, multinational, prospective, observational study that included patients, with stable chest pain or equivalent symptoms and intermediate probability of CAD, from 14 Centres in 7 European countries (http://www.clinicaltrials.gov, NCT00979199). According to the protocol, each patient underwent a coronary computed tomography angiography (CTA), stress myocardial perfusion and/or wall motion imaging. If at least one of non-invasive tests was positive, invasive coronary angiography (ICA) was performed. Blood samples were collected from patients in a fasting state during the first enrolment visit, before non-invasive imaging. Plasma aliquots were stored in the EVINCI Bio-Bank^34,35^. Patients who completed the protocol, and for whom imaging studies were submitted to the core-lab and considered of sufficient quality to be interpretable, entered a long-term clinical follow up^33^. A subset of these patients, recruited at 7 centers from 5 European Countries as part of the Simulation Modeling of coronary ARTery disease: a Tool for clinical decision support (SMARTool) study^36^, underwent a second coronary CTA scan and blood sampling at follow-up. The protocols of the EVINCI and SMARTool studies were approved by all local ethics committees, all patients gave their written informed consent to participate, and the procedures followed were in accordance with institutional guidelines. The population of the present study thus consisted of N = 355 patients with complete clinical, biohumoral and imaging profiles, of whom 154 underwent a follow-up coronary CTA. The study flow diagram is detailed in Fig. 4.

### Clinical and bio-humoral variables

Informations on cardiovascular risk factors, including age, gender, family history of CAD, smoking status, diabetes mellitus, hypercholesterolemia, hypertension, obesity, medication use and biohumoral profiles were collected. In particular, Body Mass Index (BMI) was calculated as body weight (in kg) divided by the square of the height (in m) and obesity was defined as BMI > 30 kg/m^2^. Diabetes was defined as fasting plasma glucose (FPG) > 125 mg/dL or treatment with glucose lowering medications. The homeostatic model assessment of insulin resistance index (HOMA-IR) was calculated as fasting glucose (mg/dL) × fasting insulin (pmol/L)/8.66. The presence of metabolic syndrome was diagnosed according to the National Cholesterol Education Program Adult Treatment Panel III criteria, as previously reported^37^. The TyG index^23^ was calculated with the formula ln [fasting TG (mg/dL) × FPG (mg/dL)/2].

Bio-humoral markers associated with lipid and glucose metabolism, liver function, adipose tissue function, systemic inflammation, endothelial activation and myocardial damage were determined at baseline in all patients and at follow-up in the subgroup of patients submitted to a second CTA scan. The analytical methods and ranges of normality were previously reported^32,33,36,38^. In particular, Total-C, TG, and HDL-C levels (mmol/L) were evaluated using standard methods. LDL-C was calculated using the Friedewald formula^37^. Remnant cholesterol (remnant-C) was estimated as total cholesterol minus LDL-C minus HDL-C^4s^. The TG/HDL-C ratio was calculated as TG level divided by HDL-C levels.

Medical treatments were recorded at baseline in all patients and at follow-up in the subgroup of patients submitted to a second CTA scan. Early revascularization was defined as a procedure performed within the baseline work-up, i.e. within 90 days from enrolment or 30 days from baseline ICA. Late revascularization was defined as an unplanned procedure performed at follow-up (after 90 days from enrolment).

### Non-invasive imaging

The methodology for non-invasive imaging acquisition and analysis has been previously reported in detail^32,33^. In particular, significant inducible ischaemia was defined by stress induced regional perfusion abnormalities involving ≥ 10% of left ventricular (LV) myocardium at myocardial perfusion imaging (Single Photon Emission Computed Tomography, SPECT, or Positron Emission Tomography, PET) or the equivalent > 2 segments at wall motion imaging (Echocardiography or Cardiac Magnetic Resonance, CMR)^35^. Obstructive CAD was defined at both CTA and ICA as > 50% stenosis in at least one major coronary vessel. In addition, for CTA each segment of the American Heart Association (AHA) 17-coronary segment model was assessed for interpretability, and interpretable segments were evaluated for the degree of stenosis of the coronary artery and plaque composition. If a plaque was present, plaque composition was visually determined (calcified, non-calcified, and mixed). Only one type of plaque composition could be assigned to a single segment. A comprehensive coronary atherosclerotic risk score, previously validated as a predictor of adverse events (all-cause death or non-fatal MI) in patients without known CAD, was derived from CTA scans by integration of all data on the location, severity and composition of coronary plaques^18^.

### Follow up and outcome endpoints

The clinical follow-up was performed by clinical visits and/or structured phone interviews at 3–6 months and each year after enrollment^33^.The primary outcome was a combination of major adverse events (AEs), including all-cause death and non-fatal myocardial infarction.

### Statistical analysis

Categorical variables are presented as numbers (percentage), continuous variables as mean ± standard deviation (SD). No normally distributed data were Ln transformed. For descriptive purposes, we compared means ± SD or percentages, as appropriate, across the TG/HDL-C ratio quartiles, and between the IV and the I quartile, using analysis of variance (ANOVA) with post-hoc tests for multiple comparisons or the Chi-square test, as appropriate. Pearson’s correlation was used to assess the relation between bio-humural variables and the TG/HDL-C ratio.

Survival analysis was performed using the Kaplan Meier’s method and the log-rank test from the time of enrolment until the occurrence of any component of the primary outcome. Cox models were constructed to evaluate the prognostic performance of elevated TG/HDL-C ratio (IV quartile) in predicting patient’s outcome. The effects of clinical/bio-humoral/coronary angiography variables and CTA score (considered as a synthetic variable describing the severity and extent of coronary atherosclerosis from CTA assessment) on patient outcome were considered, and Cox multivariable models were developed considering variables with a *P* value < 0.1 at univariable Cox analysis to build-up the final model (see Supplemental Tables S1, S2, S3). Thus, gender, HDL-C, TG, remnant-C, continuous values of TG/HDL-C ratio, ApoA1, the TyG index, hs-cTnT, the CTA score and obstructive CAD at ICA were sequentially included in the models and removed if they did not affect the relationship between TG/HDL-C (IV quartile) and outcome.

The comparison between clinical, bio-humoral and CTA data, obtained at the time of the first scan and of the second scan, was performed by the paired t test or χ^2^ test as appropriate, in the subpopulation of 154 patients submitted to repeated CTA and in the two subgroups defined by the TG/HDL-C ratio at baseline (below or above the median value of 2.095). To assess the relationships of TG/HDL-C ratio with the progression of the associated cardio-metabolic risk profile and of the coronary atherosclerosis risk score, the ∆ (follow-up minus baseline) values of TGs, remnant-C, TG/HDL-C ratio, TyG index, CTA score and IL-6 were also compared in the two subgroups using the Student’s t-test.

All analyses were performed using the SPSS 23 software. A 2-sided value of *P* < 0.05 was considered statistically significant.

### Ethics approval and consent to participate

The authors state that this study complies with the Declaration of Helsinki, that the research protocol has been approved by the Ethics Committee of the coordinating center (Sezione Comitato Etico Area Vasta Nord Ovest c/o Azienda Ospedaliero Universitaria Pisana, Pisa, Italy) and by the locally appointed Ethics Committees of the participating centers, and that informed consent has been obtained from the subjects (or their legally authorized representative).

## Supplementary Information

Supplementary Information.

## Data Availability

The datasets used during the current study are available from the corresponding author on reasonable request.
