# Supplementary material for: Triglycerides and low HDL cholesterol predict coronary heart disease risk in patients with stable angina
Source: Sci Rep. 2021 Oct 20;11:20714. doi: 10.1038/s41598-021-00020-3 (PMC8528835; doi:10.1038/s41598-021-00020-3)
Supplement: Supplementary file 1 — Supplementary Information. [file 41598_2021_20_MOESM1_ESM.pdf]

Supplementary Material

Figure S1: Relationship between TG/HDL-C ratio and bio-humoral variables, excluding patients with diabetes.

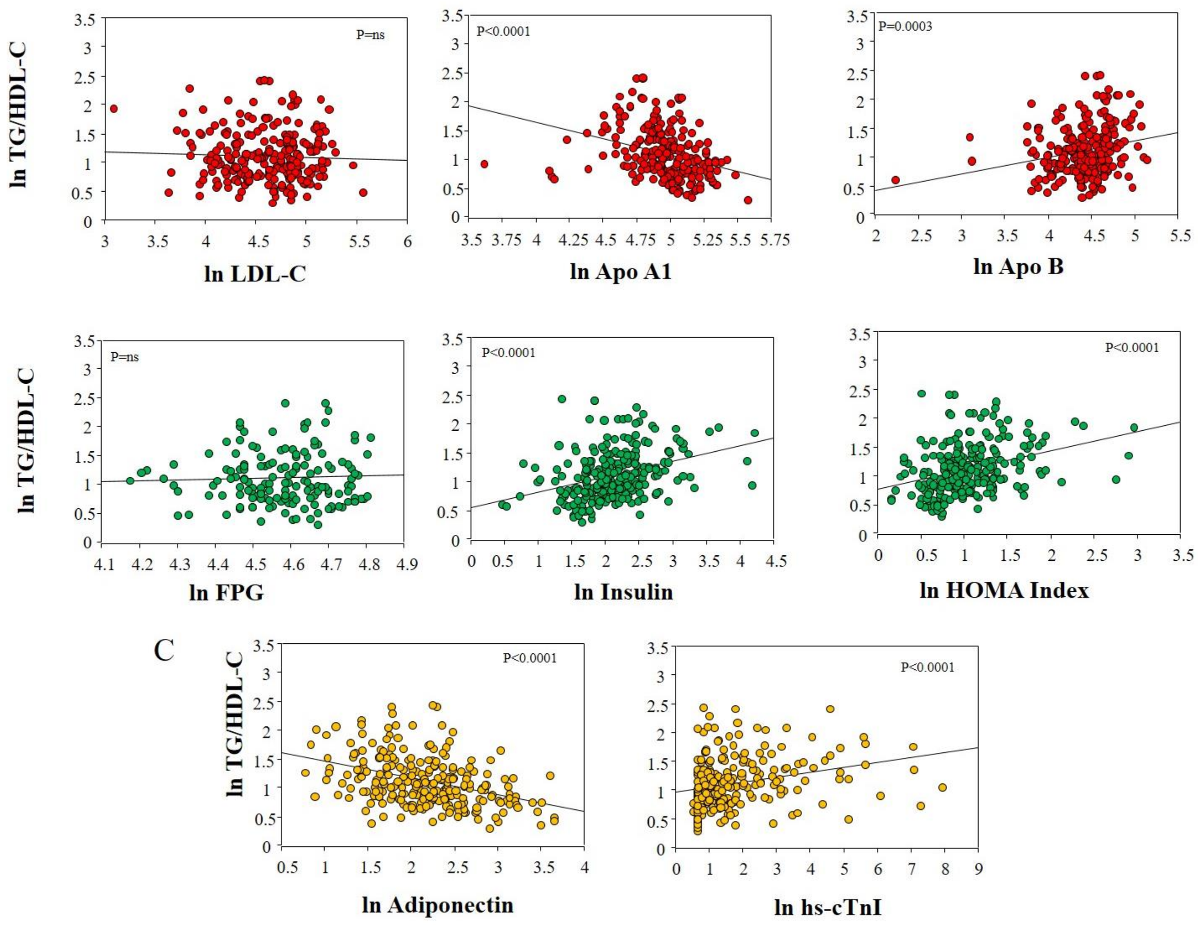

Table S1: Association of clinical variables and treatments with the composite outcome end-point at Univariate Cox analysis.

	**Univariate analysis**		
	**HR**	**95% CI**	**P value**
**Demographics**			
Age, years	1.020	0.972-1.070	ns
Male gender	2.527	1.006-6.344	0.0484
**Clinical characteristics**			
Typical angina	2.682	0.786-9.153	ns
LVEF%	0.966	0.924-1.011	ns
**Cardiovascular risk factors**			
Family history of CAD	1.134	0.489-2.630	ns
Diabetes	0.668	0.300-1.489	ns
Hypercholesterolemia	1.129	0.506-2.518	ns
Hypertension	1.189	0.534-2.647	ns
Smoking	0.615	0.256-1.482	ns
BMI, kg/m^2	0.994	0.904-1.094	ns
Metabolic Syndrome	0.561	0.256-1.230	ns
**Medications**			
Beta-blockers	0.645	0.294-1.416	ns
Calcium channel blockers	0.525	0.209-1.316	ns
ACE Inhibitors	0.734	0.324-1.665	ns
ARBs	0.684	0.272-1.720	ns
Diuretics	0.541	0.226-1.296	ns
Anti-diabetic	0.513	0.221-1.189	ns
Statins	0.602	0.259-1.398	ns
Aspirin	0.701	0.292-1.682	ns

Anti-coagulants	0.381	0.051-2.820	ns

Table S2: Association of biohumoral variables with the composite outcome end-point at Univariate Cox analysis.

	**Univariate analysis**		
	**HR**	**95% CI**	**P value**
**Lipid Metabolism**			
Total cholesterol, mg/dL	0.402	0.092-1.754	ns
LDL-C, mg/dL	0.555	0.210-1.466	ns
HDL-C, mg/dL	0.299	0.084-1.060	0.0614
Total/HDL-C	1.090	0.972-1.222	ns
Remnant-C	1.014	0.995 – 1.033	ns
Tryglicerides, mg/dL	1.870	0.942-3.713	0.0737
TG/HDL-C	1.789	1.061-3.015	0.0291
TG/HDL-C ratio (IVQ)	3.007	1.371-6.595	0.0060
Apo A1, mg/dL	0.153	0.052-0.452	0.0007
Apo B, mg/dL	0.514	0.178-1.482	ns
Apo B/Apo A1	2.857	0.651-12.542	ns
Lipoprotein (a)	0.853	0.582-1.249	ns
PCSK9, ng/mL	1.051	0.491-2.252	ns
**Glucose metabolism**			ns
FPG, mg/dL	1.670	0.411-6.792	ns
Insulin, μUI/mL	1.356	0.809-2.262	ns
HOMA-IR index	1.422	0.781-2.589	ns
TyG index	1.649	0.943-2.885	0.0794
**Hepatic function**			
AST, IU/L	1.032	0.329-3.230	ns
ALT, IU/L	0.958	0.400-2.290	ns
ALP, IU/L	2.438	0.807-7.360	ns
GGT, IU/L	1.168	0.548-2.491	ns

**Inflammation**			
hs-CRP, mg/dL	0.503	0.092-2.735	ns
Interleukin 6, ng/L	0.711	0.267-1.894	ns
**Adipocytokines**			
Leptin	0.960	0.618-1.491	ns
Adiponectin, μg/mL	0.676	0.349-1.312	ns
**Myocardial Damage**			
hs-cTnT, ng/L	1.574	0.952-2.604	0.0771
hs-cTnI, ng/L	1.146	0.904-1.452	ns
NT-proBNP, ng/L	0.959	0.674-1.364	ns

Table S3: Association of Imaging variables and invasive procedures with the composite outcome end-point at Univariate Cox analysis.

	HR	95% CI	P value
**Baseline CTA**			
Obstructive CAD (at CTA)	3.038	1.038-6.681	0.0057
Total N. of plaques	1.178	1.075-1.291	0.0005
N. of calcified plaques	1.186	1.026-1.371	0.0208
N. of mixed/non-calcified plaques	1.139	1.037-1.250	0.0063
CTA Score	1.063	1.028-1.098	0.0003
**Baseline Stress Imaging**			
Significant ischemia	1.525	0.636-3.655	ns
**Baseline Invasive Procedures**			
Obstructive CAD (at ICA)	2.275	1.018-5.083	0.0450
Early Revascularization	1.555	0.619-3.904	ns

Table S4. Comparison of clinical, bio-humoral profiles and CTA risk score from baseline to follow up in the two groups, defined by baseline TG/HDL-C ratio below (low) or above (high) the median value, from the 154 patients re-evaluated and submitted to a second CTA scan.

	Low TG/HDL-C ratio N=88			High TG/HDL-C ratio N=66		
	**Baseline**	**Follow up**	**P value**	**Baseline**	**Follow up**	**P value**
**Clinical characteristics**						
Age, years	61±8	68±8	<0.0001	61±8	67±8	<0.0001
Male gender	38 (43)	38 (43)	---	47 (71)*	47 (71)*	---
Typical angina	26 (30)	8 (9)	ns	21 (32)	5 (8)	0.0161
BMI	26.94±3.73	26.76±3.56	ns	28.31±3.30*	27.72±3.00	ns
**Medications**						
Beta-blockers	37 (42)	37 (42)	<0.0001	32 (48)	36 (55)	<0.0001
Calcium channel blockers	4 (5)	18 (20)	ns	8 (12)	16 (24)	<0.0001
ACE Inhibitors	22 (25)	34 (39)	0.0010	28 (42)*	32 (48)	0.0014
ARBs	19 (22)	14 (16)	0.0004	7 (11)	12 (18)	0.0001
Diuretics	13 (15)	12 (14)	<0.0001	14 (21)	11 (17)	0.0031
Anti-diabetic	13 (15)	25 (28)	<0.0001	15 (23)	19 (29)	<0.0001
Statins	46 (51)	52 (59)	0.0001	32 (50)	46 (70)	0.0476
Aspirin	56 (64)	53 (60)	ns	45 (68)	40 (61)	ns
Anti-coagulants	1 (1)	1 (1)	ns	0 (0)	1 (1)	--
**Lipids Metabolism**						
Total cholesterol, mg/dL	180±49	183±48	ns	181±47	172±46	ns
LDL-C, mg/dL	105±40	96±41	0.0337	108±41	91±43	0.0036
HDL-C, mg/dL	60±16	64±19	0.0201	42±11*	49±12*	<0.0001
Total/HDL-C	3.1±0.7	3.0±0.7	ns	4.4±1.2*	3.6±1.0*	<0.0001

Remnant-C, mg/dL	15±5	24±11	<0.0001	31±12*	32±20*	ns
Triglicerides, mg/dL	77±24	118±56	<0.0001	156±60*	159±81*	ns
Triglicerides/HDL-C	1.4±0.4	2.1±1.2	<0.0001	4.0±2.2*	3.5±2.3*	ns
PCSK9, ng/mL	223±123	252±84	0.0006	225±199	240±74	0.0021
**Glucose metabolism**						
FPG, mg/dL	107±23	107±23	ns	112±31	112±33	ns
Insulin, μUI/mL	7.9±5.4	9.2±9.9	ns	14.6±15.3*	13.9±12.8*	ns
HOMA-IR index	2.3±2.1	2.8±4.7	<0.0001	4.3±5.2*	4.0±4.1*	0.0004
TyG index	8.26±0.42	8.65±0.49	<0.0001	8.99±0.42*	9.02±0.63*	ns
**Hepatic function**						
AST, IU/L	24±8	24±7	ns	24±10	25±9	ns
ALT, IU/L	18±8	19±8	ns	21±9*	22±10*	ns
ALP, IU/L	48±19	53±19	ns	51±20	53±21	ns
GGT, IU/L	31±17	29±17	ns	41±16*	33±17	0.0004
**Inflammation**						
hs-CRP, mg/dL	0.39±0.85	0.20±0.22	0.0389	0.41±0.49	0.43±0.56*	ns
Interleukin 6, ng/L	1.02±1.44	1.23±0.94	0.0018	1.03±1.01	2.61±5.72*	0.0010
**Endothelial Activation**						
ICAM1, ng/mL	176±59	180±56	ns	211±34*	219±106*	ns
VCAM1, ng/mL	506±108	574±117	<0.0001	562±190*	638±199*	<0.0001
**Myocardial damage**						
hs-cTnT, ng/L	6.86±4.14	6.60±3.43	ns	8.51±6.45*	9.13±7.31*	ns
**CTA**						
CTA Score	10.11±10.34	11.71±9.46	<0.0001	12.09±10.33	15.12±9.48*	<0.0001

Continuous variables are presented as mean ± standard deviation, categorical variables as absolute N and (%)
*P<0.05 for paired comparison between the two groups of patients either at baseline or at follow-up
